# Are male nurses sexually harassed? A cross-sectional study in the Greek Health System

**DOI:** 10.1186/s12912-021-00656-6

**Published:** 2021-08-09

**Authors:** Panagiotis Papantoniou

**Affiliations:** Warwick Business School, University of Warwick, Coventry, CV4 7AL UK

**Keywords:** Sexual harassment, Male nurses, SEQ, Silence, Greece

## Abstract

**Background:**

The #MeToo and #Times Up movements have put a global spotlight on the phenomenon of sexual harassment in healthcare. Yet, most studies have explored sexual harassment among female professionals. This study departs from current research practices and investigates the frequency of sexual harassment in male nurses working in the Greek NHS and the reasons for not reporting their experiences.

**Methods:**

A cross-sectional study was conducted using the Sexual Experiences Questionnaire (SEQ) to collect data from 507 male nurses working in Greece's various settings during October and February 2021. The electronic survey was sent to male nurses (n=3,091 registered with the Hellenic Association of Nurses. Survey items were consent form, demographics, three-dimensions of sexual harassment, silencing and negative consequences. Questions were measured using five-point Likert scales, binary scale and multiple-choice questions. ANOVA and T-tests were used to investigate whether specific groups more frequently dealt with sexual harassment. Multiple regression analyses were conducted to investigate the association between independent variables (sexually harassing behaviours) and the dependent variable (participants' negative physical, mental, and job-related outcomes).

**Results:**

40% of male nurses have experienced sexual harassment at least once in their working lives, and the most common form of sexual harassment faced was gender harassment, followed by unwanted sexual attention. Male doctors and male nurses were the most common perpetrators. Private and younger male nurses with up to 5 years of experience experienced more frequent sexual harassment. 30% did not report sexual harassment due to the fear that no one would believe them, and because of beliefs, no action would be taken against the wrongdoer. Multiple regression analyses showed that unwanted sexual attention and sexual coercion were associated with physical and job-related outcomes. Cronbach Alpha was 0.91.

**Conclusion:**

A high proportion of male nurses have experienced sexual harassment during their careers. Being younger with limited working experience and working in the private sector were positively associated with sexual harassment. Policymakers and health managers should focus on sexual harassment prevention strategies and report-enabling policies.

## Background

The recent #Me Too and Time's Up movements have emphasized the ubiquity of sexual harassment [1]. According to the U.S. Equal Employment Opportunity Commission (2010), sexual harassment is any unwelcome sexual advances, requests for sexual favours, and other verbal or physical conduct of a sexual nature that explicitly or implicitly affects an individual's employment or creates an intimidating, hostile, or offensive work environment' [2]. Fitzgerald and Shullman (1990) defined sexual harassment behaviorally as three main elements: gender harassment, unwanted sexual attention, and sexual coercion [3].

A recent study by the United Nations (2016) has shown that one in three women globally has dealt with sexual harassment at least once in their lives [4]. Unsurprisingly, sexual harassment as a social phenomenon can be found in all workplaces, including healthcare. Prevalence rates of sexual harassment among nurses are high, revealing the pervasiveness of the phenomenon. For example, a recent systematic review showed that sexual harassment rates ranged from 10% to 90%, while the most common type was gender harassment [5]. Another quantitative review focusing on nurse’s exposure to physical violence revealed that approximately 1 in 4 nurses worldwide has dealt with at least one form of sexual harassment [6]. Even studies conducted in Sub-Saharan countries show that sexual harassment is a common phenomenon in nurses. Explicitly, prevalence rates varied from 10% in Gambia [7] to 16% in Ethiopia [8]

Scholars have proposed various reasons to explain the high frequency of sexual harassment in healthcare and nursing. Somani et al. (2015) suggested that because the nursing profession is predominately female-oriented, and female nurses have historically dealt with sexual harassment at work, this magnifies the problem in the workplace [9]. Despite the high frequency of sexual harassment, it has been well-found that sexual harassment is more directed towards vulnerable and economically dependent nurses, such as young nurses with limited experience. A cross-sectional study in Israel has found that nursing students are equally likely to deal with sexual harassment in clinical settings, despite that their not full-time employees [10].

As for contextual factors that stimulate sexual harassment, Frank, Brogan, & Schiffman (1998) emphasized the hierarchical structures and the authoritative leadership style of clinical settings, Berdahl (2007) focused on the role of power imbalances between male doctors holding managerial positions and female nurses, with the former using sexual harassment to reinforce their social status within the hospital [11, 12]. McLaughlin et al. (2012) discussed the role of organizational cultures that tolerate sexual harassment in clinical settings, whereas Zhang et al. (2020) argued that because healthcare settings are often isolated working environments, the prevalence of sexual harassment for healthcare workers is high [13, 14]

Sexual harassment has been shown to have adverse mental, physical and job-related outcomes for victims. The meta-analysis of Willness, Steel, and Lee (2007), as well as the systematic review of Kahsay et al. (2020), have shown that sexual harassment is linked to decreased job satisfaction, commitment, productivity, stress, burnout and turnover [15–17]. As for the psychological consequences, the review article of McDonald (2012) and the meta-analysis of Zeng et al. (2019) have illustrated that sexual harassment causes stress, hypervigilance, psychological burnout, avoidance and depression [6, 18, 19]. Expected physical outcomes of sexual harassment are headaches, exhaustion, nausea, sleep difficulties, suicidal behaviour and menstrual disturbances [20, 21].

The above reveals that sexual harassment is a severe and common phenomenon in nursing and generally healthcare. Although the predominant attention has been given towards exploring sexual harassment among female nurses, there have been limited articles investigating the frequency of phenomenon among male nurses. Although empirical evidence suggests that men are the most common perpetrators, they can also be victims of sexual harassment, especially within female-dominated environments and workgroups. Additionally, current knowledge is limited to the frequency of sexual harassment among male nurses. We know nothing of whether male victims report their experiences and, if they do not, why they do so. This study aims at filling this critical gap and investigate the prevalence of sexual harassment of male nurses in the Greek Health System, the frequency of reporting sexual harassment and the reasons of male victims for not reporting their experiences.

Based on the findings, multiple contributions are offered. First, the extent of understanding of sexual harassment has been broadened by revealing the high frequency of sexual harassment among male nurses. Second, this study illustrates the frequency of gender harassment, unwanted sexual attention, and sexual coercion, which constitute sexual harassment. Third, we highlight that sexual harassment of male nurses is crucially underreported; that is why there is a misconception that male nurses are not sexually harassed. The study reveals various reasons why male victims do not report sexual harassment, with the most prevalent being feelings that no one would believe their allegations. Taking these contributions together, we heed and echo calls to better and holistically contextualize sexual harassment specifically and other forms of workplace mistreatment and discrimination against men broadly

### Aim and objectives

The aim of this study was to explore the frequency of sexual harassment among male nurses, the types of sexual harassment, perpetrators and its physical, mental and job-related adverse outcomes of male nurses working in hospitals. An additional goal is to identify the prevalence of reporting sexual harassment, and in the case of not reporting, the main reasons driving this decision. Last, one last objective was to understand the association of variables affecting sexual harassment prevalence among male nurses.

## Methods

### Study design and participants

An electronic-based cross-sectional study was conducted between October 2020 and February 2021 among male nurses working in Greece's various health facilities. Convenience sampling was used due to the sensitive nature of the research topic based on respondents' willingness and availability. The online survey was uploaded to the official Facebook page of the Hellenic Association of nurses. The link was also forwarded to more male nurses through other professional Facebook pages on Nursing in Greece. Participants who completed the survey were kindly asked to forward the survey link to as many male nurses as possible. Hence, the link of the e-survey was rolled out to male nurses apart from the first point of contact. The link was accessed by 3,091 male nurses, of which 507 completed the survey for a response rate of 18.44%. On clicking the link, respondents were directed to the first page, which encompassed the researcher's information, the study's goal, how data would be managed, and participants' right to withdraw from the study whenever they wanted. It was also stated that the completion of the survey would take approximately 15 minutes and participation in this study was entirely voluntary while to proceed to the questions part, participants were asked to give their consent and allow their data to be used for research purposes. The target population was male nurses working in the Health sector of Greece. The inclusion criteria for participating in the study were: (a) having worked in the Greek NHS, (b) have a nursing degree.

To increase the electronic survey response rate, we used two evidence-based strategies [22]. Throughout the data collection period, we posted four messages on the professional Facebook page of the Hellenic Association of Nurses and communicated regularly with the representatives of the association to ask them to encourage more nurses to fill the questionnaire. Last, to prevent participants from completing the questionnaire multiple times, we enabled the 'Prevent Ballot box' from Qualtrics that does not let people with the same IP respond many times.

### Data collection instrument

The survey was developed by the principal author in collaboration with partners from the Hellenic Nurse Association. All the research team members first reviewed and revised questions to ensure clarity and alignment of questions with the leading research goals. The draft survey was then shared with five academic experts in Nursing and Nursing Management to request feedback on content, simplicity and significance. Following the experts' suggestions, four additional questions were included, while the framing of ten questions was amended to enhance clarity. Before piloting, the usability and technical functions of the online study was tested. The final survey was pilot tested with 20 male nurses working in various contexts and not involved in designing the survey. The final survey was administered in the Greek language and comprised four sections with 35 total questions. The first section included the study purpose, researchers’ contact details and informed consent that needed to be signed before moving to the following parts of the survey. The second part asked about respondents information such as gender, age, education, employment, and years of work experience. The third part included questions measured on a 5-point Likert scale (1: very often, 2: often, 3: occasionally, 4: rarely, 5: never) to describe how often male nurses experienced sexual harassment. The latter was investigated using the Sexual Experiences Questionnaire (SEQ), which is the most reliable questionnaire of sexual harassment [23, 24]. The questionnaire follows a three-factor structure with a total of 22 questions that explore the frequency of gender harassment, unwanted sexual attention and sexual coercion in a 5-Point Likert scale (1: very often, 2: often, 3: occasionally, 4: rarely, 5: never). Participants were presented with the following question: "While working as a nurse, how often have you been in a situation where someone has:" and then questions appeared. The fourth and final part included three multiple-choice questions investigating the type of wrongdoer (male doctor, male nurse, female doctor, female nurse, visitor, patient), the place where sexual harassment occurred, and the main reasons why victims did not report their experiences. There were also two questions measured in a binary format (Yes/No) that examined whether victims reported their experiences as well as whether the hospital conducted an internal investigation. These questions were asked to achieve a holistic understanding of participant’s experiences with sexual harassment in healthcare settings and understand how hospitals dealt with victim’s complaints.

### Scoring the SEQ

There are various ways of scoring the SEQ. In this article, we used the option proposed by Koss et al. (2007) in which the frequency of each sexual harassment behaviour was derived by subtracting the frequency of victims that had never experienced the particular behaviour during their working lives [25]. Following this approach, the frequency of all sexual harassment behaviours was calculated, while to find the aggregate frequency of sexual harassment, we calculated the average of all frequencies produced in the first scoring step. To evaluate the reliability of the questionnaire, Cronbach’s Alpha was calculated. The latter is an estimator of internal consistency, which shows how closely related a set of survey questions are as a group [26]. Cronbach’s alpha coefficient value for gender harassment questions was 0,87, the exact figure for Unwanted Sexual Attention questions was 0,87, while for sexual coercion questions was calculated at 0,86. The whole scale Cronbach’s alpha was 0,915.

### Data analysis

We used the Statistical Package for Social Sciences (SPSS) version 27. Firstly, Cronbach's alpha was calculated and checked whether scale variables were normally distributed using the Shapiro-Wilk test. Descriptive statistics were performed to describe the demographic characteristics and the main sexual harassment variables. Independent sample T-tests were used to investigate whether male nurses that worked in a particular sector more frequently experienced sexually harassing behaviour. One-way ANOVA was conducted to explore the relationship between ordinal demographic variables (work experience, age group) with scale variables. Backward multiple regression analyses were performed to determine which behaviours were associated with participant's negative physical, mental and job-related consequences. All SEQ variables were included in the regression model, while the backward regression approach produced the regression model which best explained the data. A p-value of less than 0.05 was considered statistically significant.

### Ethical considerations

The Humanities & Social Sciences Research Ethics Committee (HSSREC) of Warwick University approved the study (E-645-01-20). All participants provided informed consent. Participant involvement was voluntary, and those who were unwilling to continue and quit any stage of the process were able to do so without any restriction. Data were carefully analyzed to ensure the anonymity, privacy and confidentiality of the participant nurses.

## Results

### Sample characteristics

Table 1 illustrates that most male nurses worked in the Public sector (87%), belonged in the age group 31-40 (38%), hold a degree from a Technological Educational Institute (38%). The majority had up to five years of experience (38.5%), while almost one in two was unmarried.

**Table 1:** Demographic data

Variables	Sample (n=507)	Percentage (%)
**Age**		
18-30	182	35.9
31-40	195	38.5
41-50	130	25.6
51-60	-	-
**Educational level**		
Secondary education	52	10.3
Vocational training	130	25.6
Technological education	195	38.5
University education	65	12.8
Master's	65	12.8
PhD		
**Employment sector**		
Public	442	87.2
Private	65	12.8
**Work experience**		
Less than a year	52	10.3
1-5 years	195	38.5
6-10 years	180	35.50
11-16 years	70	13.80
16+ years	10	1.9
**Marital status**		
Married	195	38.5
Unmarried	247	48.7
Divorced	65	12.8

### Sexual experiences

Table 2 shows that approximately 4 in 10 male nurses have experienced sexual harassment at least once in their working lives (923.24/22). The most frequent form of sexual harassment is gender harassment (62%), followed by unwanted sexual attention (48%) and sexual coercion (24%). Most sexually harassing behaviours occasionally occur, except for some unwanted sexual attention actions that happen very often and often.

**Table 2:** Sexual Experiences results

Variables	Very often (n, %)	Often (n, %)	Occasionally (n, %)	Rarely (n, %)	Never (n, %)	Frequency (%)
**Gender harassment**						
Has treated you differently because of your sex	-	-	195 (38.5)	117 (23.1)	195 (38.5)	61.5
Displayed, used, or distributed sexist or suggestive materials	-	-	65 (12.8)	195 (38.5)	247 (48.7)	51.3
Made offensive sexist remarks	-	-	130 (25.6)	247 (48.7)	130 (25.6)	74.4
Put you down or was condescending to you because of your sex	-	-	50 (9.86)	262 (51.67)	195 (38.46)	61.54
**Unwanted sexual attention**						
Repeatedly told sexual stories or jokes that were offensive to you	-	52 (10.3)	130 (25.6)	65 (12.8)	260 (51.3)	48.7
Whistled, called, or hooted at you in a sexual way	65 (12.8)	35 (6.9)	147 (28.99)	65 (12.8)	195 (38.5)	61.5
Made unwelcome attempts to draw you into a discussion of sexual matters	65 (12.8)	24 (4.73)	106 (20.9)	117 (23.1)	195 (38.5)	61.5
Made crude and offensive sexual remarks, either publicly			130 (25.6)	52 (10.3)	325 (64.1)	35.9
Made offensive remarks about your appearance, body, or sexual activities	-	-	12 (2.36)	170 (33.53)	325 (64.1)	35.9
Made gestures or used body language of a sexual nature which embarrassed or offended you	65 (12.8)	11 (2.16)	41 (8)	130 (25.6)	260 (51.3)	48.7
Stared, leered, or ogled you in a way that made you feel uncomfortable	-	-	65 (12.8)	312 (61.5)	130 (25.6)	74.4
Has he made unwanted attempts to establish a romantic sexual relationship with you despite your efforts to discourage it?	-	-	65 (12.8)	195 (38.5)	247 (48.7)	51.3
Continued to ask you for dates, drinks, dinner, etc., even though you said "No"	-	-	65 (12.8)	130 (25.6)	312 (61.5)	38.5
Touched you in a way that made you feel uncomfortable	-	-	-	130 (25.6)	377 (74.4)	25.6
**Sexual coercion**						
Made unwanted attempts to stroke. fondle, or kiss you	-	-	-	130 (25.6)	377 (74.4)	25.6
Attempted to have sex with you without your consent or against your will, but was unsuccessful	-	-	65 (12.8)	130 (25.6)	312 (61.5)	38.5
Had sex with you without your consent or against your will	-	-	-	1 (0.19)	506 (99.80)	0.20
He/she made you feel like you were being bribed with some reward or special treatment to engage in sexual behaviour	-	-	65 (12.8)	130 (25.6)	312 (61.5)	38.5
He/she made you feel threatened with some retaliation for not being sexually. cooperative	-	-	13 (2.56)	52 (10.25)	442 (87.2)	12.8
Treated you badly for refusing to have sex	-	-	65 (12.8)	130 (25.6)	312 (61.5)	38.5
Implied faster promotions or better treatment if you were sexually cooperative	-	-	42 (8.28)	88 (17.35)	377 (74.4)	25.6
It made you afraid you would be treated poorly if you didn't cooperate sexually	-	-	-	65 (12.8)	442 (87.2)	12.8

As for the perpetrators, most of them were male doctors (38.4%), followed by male nurses (24%), female doctors (18%), patients (11%) and patient's family member (9%). Most cases occurred in the hallway (50%), patient care units (25%) and surgery areas (17%). In most cases, victims ignored the behaviour and avoided the harasser (45%), stayed silent (30%), and only 1 in 4 told the harasser to stop. In the cases where victims reported the incident, the organization did not initiate an internal investigation (99%), whereas when it did, the perpetrator was not penalized in all cases.

### Silencing of male nurses

Participants were asked to articulate the main reasons why they did not report sexual harassment. Explicitly, the reasons can be grouped into four categories; the first and most prevalent concerns victim's fear that no one would believe his allegations (40%), the second relates to the fear of being labelled negatively by colleagues at work (20%), the third concerns the fear of being accused of overreacting (14%) and the fourth concerns victim's beliefs that no action would be taken against the perpetrator (25%).

### Negative consequences of sexual harassment

35.9% of victims experienced physical problems, with the most frequent issues being (sleep difficulties and engaging in unhealthy behaviours such as drinking alcohol and smoking). Three in ten victims dealt with mental issues due to sexual harassment, such as alienation from others at work and stress being the most common problems. Last, 18% of the respondents reported that their ability to do their job was negatively affected by unnecessary sick leaves and difficulties, with concentration being the most frequent job-related issues faced by victims.

### T-tests between employment sector and sexual harassment behaviours

T-tests showed that private nurses compared to public nurses, more frequently experienced sexually harassing behaviours. On the contrary, public nurses compared to private nurses more frequently dealt with sexual comments and were made unwanted attempts to have sex against their will.

### ANOVA between age and sexual harassment behaviours

The analysis ANOVA between nurses age group and sexual harassment behaviours showed statistically significant differences that were particularly evident when comparing the 18-30 with the 31-40 and 41-50 age groups. Post hoc tests (LSD) showed that younger male nurses more frequently dealt with gender harassment, unwanted sexual attention, and sexual coercion.

### ANOVA between work experience and sexual harassment behaviours

The analysis ANOVA showed that years of experience is a crucial factor influencing the frequency of sexual harassment. Specifically, those with up to 5 years of experience (<1 year of experience and 1-5) more frequently dealt with sexual harassment than those with more than five years of experience. Post hoc tests (LSD) revealed that those with 1-5 years of experience more frequently faced gender harassment and sexual coercion, whereas those less than a year of experience dealt with unwanted sexual attention more regularly.

### Multiple regression analysis between sexual harassment and negative consequences

#### Physical consequences

Table 3 reveals that three independent variables; offensive, sexist remarks, unwanted attempts for sex against nurse's consent, and offers for better treatment in exchange for sexual cooperation, had a statistically significant relationship with the variable exploring the magnitude of the adverse physical outcomes of sexual harassment (*R*^2^= 52%, p value<0.01)

**Table 3:** Full data from predictors identified with statistical significance on multiple regression analysis according to the outcome variable Negative Physical consequences

Outcome-Negative physical consequences, score 1-5 (linear regression)					
Predictor variables in the model	Coef.	Std. error	P value	95% Confidence Interval	
				Lower	Upper
Offensive sexist remarks	2.500	0.157	<0.001	-2,500	2,500
Attempts for unwanted sex	2.475	0.054	<0.001	-2,475	2,475
Implied faster promotions if you were sexually cooperative	2.232	0.111	<0.001	-2,232	2,232
(Constant)	5.705	0.157	<0.001	-1,500	5,705

*R*^2^: 0.52 (Adjusted *R*^2^: 0.48)

#### Mental consequences

Table 4 shows that four independent variables; mistreating and ignoring because of your sex, crude and offensive sexual remarks, gestures and offensive language of sexual nature and behaviours aiming at intimidating the victim due to his refusal to cooperate sexually, had a statistically significant relationship with the variable exploring the magnitude of the adverse mental outcomes of sexual harassment (*R*^2^= 41%, p value< 0.01)

**Table 4:** Full data from predictors identified with statistical significance on multiple regression analysis according to the outcome variable negative Mental consequences

Outcome-Negative mental consequences, score 1-5 (linear regression)					
Predictor variables in the model	Coef.	Std. error	P value	95% Confidence Interval	
				Lower	Upper
Mistreating and ignoring you because of your sex	0.232	0.066	<0.001	-0.232	0.232
Crude and offensive sexual remarks	1.375	0.048	<0.001	-1.375	1.375
Gestures and body language of a sexual nature	1.250	0.107	<0.001	-1.250	1.250
Made you afraid you would be treated poorly if you did not cooperate sexually	3.750	0.060	<0.001	-3750	3.750
(Constant)	6.041	0.318	<0.001	-6.041	6.041

*R*^2^: 0.57 (Adjusted *R*^2^: 0.51)

#### Job-related consequences

Table 5 illustrates that four independent variables; unwanted sexual touches, sexual stories, unwanted attempts to be kissed, and threats of retaliation for not being sexually cooperative, had a statistically significant relationship with the variable exploring the magnitude of the negative mental outcomes of sexual harassment (*R*^2^= 45.2%, p value< 0.01).

**Table 5:** Full data from predictors identified with statistical significance on multiple regression analysis according to the outcome variable negative job-related consequences

Outcome-Negative mental consequences, score 1-5 (linear regression)					
Predictor variables in the model	Coef.	Std. error	P value	95% Confidence Interval	
				Lower	Upper
Mistreating and ignoring you because of your sex	2.500	0.072	<0.001	-2.500	2.500
Crude and offensive sexual remarks	0.625	0.112	<0.001	-0.625	0.625
Gestures and body language of a sexual nature	1.250	0.07	<0.001	-1.250	1.250
Made you afraid you would be treated poorly if you did not cooperate sexually	3.750	0.076	<0.001	-3750	3.750
(Constant)	7.125	0.134	<0.001	-7.125	7.125

*R*^2^: 0.452 (Adjusted *R*^2^: 0.41)

## Discussion

This study is the first of its kind to provide evidence of the frequency of sexual harassment against male nurses working in Greece. The findings show that four in ten male nurses have experienced sexual harassment at least once in their working lives, revealing that rates are high. The frequency of 40% is significantly higher than the 7,8% frequency identified by other studies exploring male nurses and male nursing students in Sub-Saharan Africa [21]. This is partly because the sample was crucially higher in this study than that used in Sub-Saharan Africa, the cultural differences between the two settings and the different instruments used. The higher prevalence rate of this study compared to other similar studies can be explained by the fact that this study adopted the tripartite definition of sexual harassment (gender harassment, unwanted sexual attention and sexual coercion) while other similar studies adopted different definitions.

The most frequent type of sexual harassment faced by male nurses was gender harassment, followed by unwanted sexual attention while only a minority experienced sexual coercion. This finding is consistent with insights produced from a recent meta-analysis that showed that gender harassment against nurses is the most common form of sexual harassment, followed by unwanted sexual attention and sexual coercion [5, 27]. As for the perpetrators, the most common were male physicians and male nurses, which is consistent with most studies in sexual harassment in nurses suggesting that men are the most frequent perpetrators [28, 29].

The statistical analysis showed a statistically significant relationship between age, limited years of experience and being in the private sector with an increased prevalence of sexual harassment among male nurses. That is consistent with other studies investigating female nurses that show that young age and limited years of experience are crucial factors that increase the frequency of sexual harassment [30, 31]. Almost 36% of our respondents experienced physical problems, while 30% dealt with mental issues due to sexual harassment. However, these figures are not in line with previous systematic reviews that consider sexually harassed female victims are experiencing predominately mental health problems compared to physical health problems [5]. This finding reveals that male nurses experience sexual harassment differently compared to female nurses, with the former mainly dealing with physical health problems while the latter experiencing mental health problems.

### Limitations

Inevitably this study has limitations. First, only male nurses were surveyed while other female nurses or other physicians/professionals were not included. Secondly, the relatively low response rate (18%) may be a source of selection bias. Third, self-reported data from electronic surveys can not be confirmed, and there is a possibility that responses may be influenced by social desirability bias. Hence, the results need to be considered with caution when generalized to Greece's entire male nurse population.

### Implications for research and practice

We now turn to consider the implications of our study for research and practice. Policymakers and hospital managers should implement drastic measures to decrease the frequency of sexual harassment of male nurses in healthcare. This can be done through opening more channels for victims to report their experiences, redesigning hierarchical organizational structures that impede upward communication, creating a well-defined policy that encapsulates what sexual harassment is what is the process of reporting, establish an independent committee to assess the complaints of victims and ensure the transparency and confidentiality of the process.

Further studies are needed towards exploring what prompts sexual harassment using poststructuralist accounts of power that go beyond stable contextual variables that precipitate sexual harassment (climate, structure, power differences). Research studies should investigate sexual harassment among various health professionals and understand the dynamics of the phenomenon between different working groups. Finally, research papers should explore observer reactions and how third-party actors perpetuate sexual harassment in the workplace and indirectly force victims to stay silent. Last but not least, it is crucial to explore various forms of workplace mistreatment in healthcare and broaden the term sexual harassment to encompass more behaviours that can be considered sexual harassment.

## Conclusions

This study's results reveal that a significant proportion of male nurses working in the Greek NHS had experienced various forms of sexual harassment. Male doctors and male nurses are sexually harassing male nurses. More than a third experienced negative physical and mental health consequences, emphasizing the detrimental impact of sexual harassment on nurses' well-being. Male nurses did not report their experiences because no one would take their experiences seriously. It is recommended policymakers introduce work ethics and training programs to prevent sexual harassment in clinical settings. Nursing Associations can play a central role by proposing workplace safety policies to create a safe and secure environment in which sexual harassment is prohibited and not tolerated.

## Data Availability

The dataset used during the current study is available from the author upon reasonable request.
